# Impact of Spinopelvic sagittal alignment on the surgical outcomes of dropped head syndrome: a multi-center study

**DOI:** 10.1186/s12891-020-03416-w

**Published:** 2020-06-15

**Authors:** Yoshifumi Kudo, Tomoaki Toyone, Kenji Endo, Yuji Matsuoka, Ichiro Okano, Koji Ishikawa, Akira Matsuoka, Hiroshi Maruyama, Ryo Yamamura, Haruka Emori, Soji Tani, Toshiyuki Shirahata, Chikara Hayakawa, Yushi Hoshino, Tomoyuki Ozawa, Hidekazu Suzuki, Takato Aihara, Kazuma Murata, Taichiro Takamatsu, Katsunori Inagaki

**Affiliations:** 1 Department of Orthopaedic Surgery, Showa University, 1-5-8 Hatanodai Shinagawa-ku, Tokyo, 142-8666 Japan; 2 Department of Orthopedic Surgery, Tokyo Medical University, 6-7-1 Nishishinjuku, Shinjuku-ku, Tokyo, 160-0023 Japan

**Keywords:** Dropped head syndrome, Chin-on-chest deformity, Sagittal vertical axis, Compensatory function, Surgical outcome, Spinopelvic sagittal alignment

## Abstract

**Background:**

Most of the previous studies about the surgical treatment of dropped head syndrome (DHS) are small case series, and their primary outcome measures were cervical alignment parameters. Therefore, little is known about the associations between pre- and postoperative global sagittal alignment in the whole spine and the clinical outcomes of the surgical treatment of DHS. In this study, we investigated the surgical outcomes of DHS, including correction of cervical and global spinal sagittal alignment.

**Methods:**

This study was a retrospective observational study. Fifteen patients with DHS who had undergone correction surgery were enrolled. Surgical outcomes, including complications and implant failures, were investigated. We assessed cervical alignment parameters as well as spinopelvic global alignment parameters, including pelvic incidence (PI), lumbar lordosis (LL), and C7-sacral sagittal vertical axis (SVA). We examined the changes in these parameters using pre- and posoperative whole spine lateral radiographs. The parameters were compared between the failure and nonfailure groups.

**Results:**

Recurrence of sagittal imbalance and horizontal gaze difficulty was observed in 6 cases (40%). In all, 3 cases (20%) exhibited a distal junctional failure and required multiple surgeries with extension of fusion. Of all the radiographic parameters compared between the failure and nonfailure groups, significant differences were only observed in pre and postoperative SVA and PI-LL.

**Conclusions:**

Our results suggest that the global sagittal alignment parameters, including PI-LL and SVA, were different between the patients with failure and non failure, and these parameters might have notable impacts on surgical outcomes. Surgeons should consider PI-LL and SVA while determining the surgical course for patients with DHS.

## Background

Dropped head syndrome (DHS) is characterized by severe neck extensor weakness resulting in chin-on-chest deformity [1–3]. Various types of neurological or medical conditions can cause DHS, including neurodegenerative diseases, myopathies, sarcopenia associated with aging, and iatrogenic causes [4–8]. However, DHS can also occur without specific underlining neurological/muscular conditions [9, 10].

Patients with DHS experience various disabilities, including horizontal gaze difficulty and dysphagia, and these symptoms lead to deterioration of activity of daily living. Because DHS is a relatively rare condition, its treatment strategy has not yet been established. For severe deformity cases, surgical treatments are commonly indicated; however, studies regarding surgical treatment are mainly limited to small case series and focused on cervical alignment improvements [11–13]. Several studies have demonstrated that patients with DHS have characteristic patterns of global spinal sagittal alignment. One study reported that there was appositive correlation between C2–7SVA and upper thoracic kyphotic angle (T1-4TK) [14]. Another study reported that DHS patients can be classified into two distinct types: SVA+ and SVA- types, based on the global alignment [15]. Currently, knowledge about the influence of pre/postoperative global sagittal alignment on the outcomes of surgically treated DHS is limited.

The present study investigated the clinical outcomes, including correction of cervical and global spinal sagittal alignment, in patients with surgically treated DHS.

## Methods

### Patients and outcome measures

Patient baseline data of this particular study was derived from a multi-center study database, including DHS with various background conditions and treatments [16]. We retrospectively reviewed the records of DHS patients whose surgery was performed between 2014 and 2018. The Ethical Board approval (No.2018–2682) was obtained and consent from each patient was waived due to the retrospective and anonymous nature of this study. We defined DHS as meeting all following criteria: 1) typical chin-on-chest deformity with horizontal gaze difficulty during non-labored standing, 2) difficulty in maintaining the head upright over a few seconds, and 3) mobile deformity at least partially correctable in supine position. The criteria for surgery were: 1) the patients had persistent daily-life disability due to DHS despite at least 6 months of conservative treatment including physical therapy and neck collar, 2) the patients wanted and agreed to receive the surgical treatment, and 3) the patients were medically cleared for correction surgery. In this study, we focused on the outcomes of cervicothoracic correction surgery for idiopathic DHS cases and patients with rigid deformity due to ankylosing pathologies, including longitudinal ligament ossifications or ankylosing spondylitis, and patients with a known history of neuromuscular diseases, including Parkinson’s disease, amyotrophic lateral sclerosis, myasthenia gravis, and polymyositis, or iatrogenic causes were carefully excluded. Also, patients who underwent only thoracolumbar surgery for this pathology were excluded. The minimum postoperative follow-up period was set as 12 months.

We defined treatment failure as recurrence of sagittal imbalance/horizontal gaze difficulty that required additional surgery. If a patient refused further surgeries or his/her medical conditions did not allow additional intervention, we categorized the patient as “recommendation” and analyzed as in the failure group. The reasons for additional surgeries and major medical complications were also documented. As potential explanatory factors, age, sex, surgical approach, range of fused levels, and pre/postoperative sagittal alignment parameters were included and compared between the failure and nonfailure groups.

### Radiographic assessment of sagittal alignment parameters

For radiographic assessments, the following parameters were assessed: C2–7 angle (C2–7A), measured by the C2–7 posterior tangent method [17]; C2–7 sagittal vertical axis (C-SVA), defined as distance between the plumb line dropped from the centroid of C2 and C7; T1 slope (T1S); C7-sacral SVA (SVA), defined as distance between the plumb line dropped from the center of C7 and the posterior edge of the sacral endplate; and commonly used spinopelvic parameters such as T4-T12 thoracic kyphosis (TK), lumbar lordosis (LL), pelvic tilt (PT), and pelvic incidence (PI). We examined the changes in these parameters between pre- and postoperative whole spine lateral radiographs. These radiographs were obtained in the most stable and relaxed standing position with possible knee extension while maintaining a horizontal gaze in the fists-on-clavicle posture to ensure reasonable reproducibility [18]. All radiographic assessments were manually performed blindly to the outcomes and were conducted by two board-certified orthopedic spine surgeons using a picture-archiving and communication system (INFINITT PACS®, NFINITT Healthcare Co., Ltd., Seoul, Korea).

### Statistical analysis

Statistical analyses were performed using the paired t-test for comparisons between pre- and postoperative sagittal alignment parameters. For comparisons of sagittal alignment parameters between the failure and nonfailure groups, the Mann–Whitney U test was used since the parameters were non-normally distributed continuous variables. All statistical analyses were performed with the JMP software package (version 10.0, SAS Institute Inc., Cary, NC, USA). Statistical significance was set at *p* < 0.05.

## Results

### Demographics

**Fig. 1:**
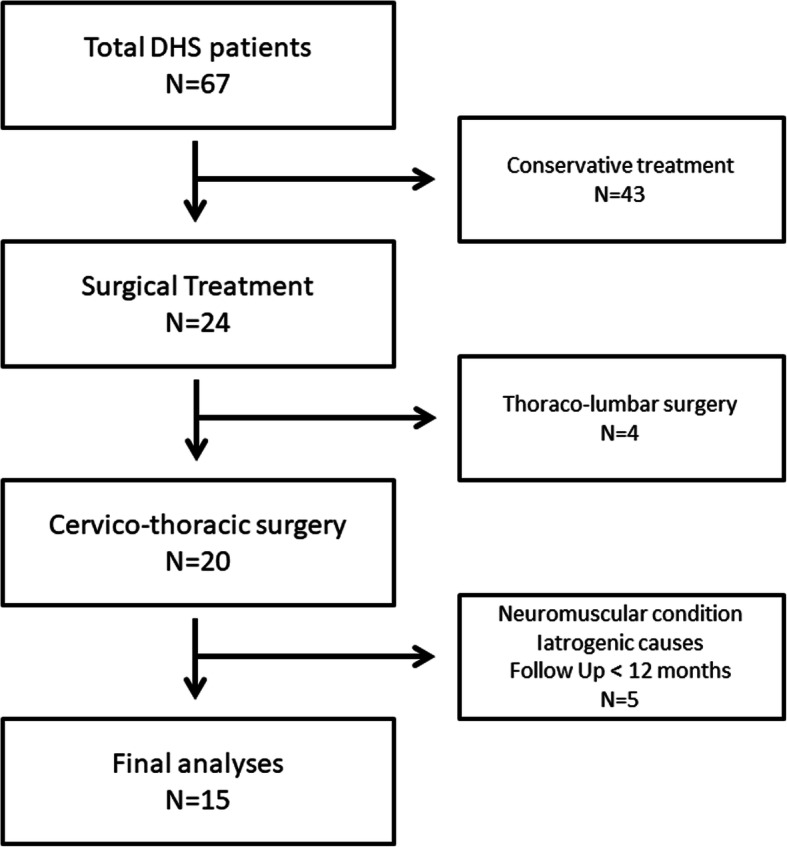
Application of exclusion criteria

Among 67 patients with DHS from multicenter database, 24 patients underwent surgery. Among them, 4 patients were excluded owing to thoracolumbar surgery and 5 patients were excluded because of iatrogenic causes, neuromuscular conditions, and lack of postoperative follow-up period. A total of 15 patients with cervicothoracic surgery were included in the final analysis (Fig. 1). The mean age was 72.1 years, and 13 patients (86.7%) were female. Average follow-up period was 26.5 months (range, 12–45 months). Anteroposterior combined approaches were used in all patients. The median [range] number of fused levels at the primary surgery was 8 [5–11] levels. No patient underwent three-column osteotomy. Patient demographics along with the preoperative sagittal alignment parameters are summarized in Table 1.

**Table 1:** Patient demographics, sagittal balance parameters, and clinical outcomes

Case	Age (y)	Sex	C2–7SVA	C2–7A	T1S	SVA	TK	LL	PI	PI-LL	PT	Approach	Levels of anterior surgery	Levels of posterior surgery	Number of fused levels	Failure	Revision surgery due to DJK	Distant lumbar surgery	Other perioperative complications
1	50	F	55	−60	23	−47	33	50	34	−16	3	AP	C4–6	C2–T2	8	–	–	–	–
2	73	F	72	−75	45	51	33	32	42	10	20	AP	C4–6	C2–T3	9	+	+	–	–
3	68	F	72	−55	43	86	17	−8	51	59	52	AP	C3–7	C3–T4	9	+	–	+	–
4	76	M	73	−19	41	−16	48	60	55	−5	22	AP	C3–6	C3–T3	8	–	–	–	–
5	80	F	53	−70	26	− 58	45	56	40	−16	4	AP	C4–7	C3–T3	8	–	–	–	–
6	85	F	54	−3	45	0	57	50	45	−5	24	AP	C3–7	C3–T1	6	–	–	–	Respiratory distress
7	79	M	66	−20	31	−11	48	59	57	−2	21	AP	C3–6	C2–T2	8	+	+	–	Severe dysphagia
8	84	F	83	−63	65	70	67	76	76	0	27	AP	C3–6	C2–T5	11	+	+	–	Deep infection
9	57	F	78	−61	52	−23	32	60	60	0	29	AP	C3–6	C3–T4	9	–	–	–	–
10	41	F	86	−24	46	−76	53	63	42	−21	16	AP	C5–7	C3–T3	8	–	–	–	–
11	81	F	44	−53	10	12	28	3	46	43	41	AP	C4–7	C3–7	5	+	–	+	–
12	80	F	49	−55	11	0	20	21	48	27	38	AP	C4–7	C2–7	6	+	–	+	–
13	82	F	78	−71	27	−11	36	23	55	22	48	AP	C3–6	C2–T3	9	–	–	–	–
14	83	F	40	−8	29	−16	62	54	40	−14	20	AP	C4–7	C4–T1	5	–	–	–	–
15	63	F	45	−72	10	−72	23	60	54	−6	18	AP	C3–6	C2–T3	9	–	–	–	–

*AP* Antero–posterior method, *DJK* distal junctional failure, *F* female, *M* male

### Clinical outcomes

Clinically significant recurrence of sagittal imbalance/horizontal gaze difficulty was observed in 6 patients (40%). Among these 6 patients, 3 (20%) had a distal junctional failure and required multiple surgeries with distal extension of fusion. Eventually, the lowest instrumented vertebra at the last follow-up was T4 in 1 and L2 in 2 patients. The remaining 3 patients (20%) demonstrated clinically significant symptomatic and/or progressive lumbar kyphosis after cervical corrective surgery. Surgical treatment of distant lumbar deformity was recommended for all these 3 patients and 2 received the treatment.

The following perioperative complications were observed: respiratory distress due to airway swelling, which required a tracheostomy in 1 patient; severe postoperative dysphagia, which required tubal feeding in 1 patient; and deep infection in 1 patient (Table 1). No neurological complications were observed. At the last follow-up, the horizontal gaze difficulty/sagittal imbalance was improved in all the patients, except for 1 who refused the additional lumbar surgery.

### Radiographic assessments

The comparisons of pre- and postoperative parameters are presented in Table 2. Significant changes were observed in C-SVA, C2–7A, SVA, and PT. The results indicate significant improvement in cervical sagittal alignment from the chin-on-chest deformity.

**Table 2:** Comparison of preoperative and postoperative parameters

	Preoperative	Postoperative	*P* value
**C-SVA**	**63.2 ± 15.4**	**34.3 ± 19.3**	< 0.001*
**C2–7A**	**−47.3 ± 25.1**	**15.3 ± 14.2**	< 0.001*
T1 slope	33.6 ± 16.4	37.5 ± 13.1	0.11
**SVA**	**−7.4 ± 47.8**	**22.1 ± 40.8**	0.007*
TK	40.1 ± 15.5	39.9 ± 14.0	0.90
LL	43.9 ± 24.2	43.3 ± 24.6	0.67
PI-LL	5.8 ± 23.8	6.5 ± 23.0	0.55
**PT**	**25.5 ± 14.2**	**22.8 ± 14.0**	0.04*

*Significantly different

The comparison of sagittal alignment parameters between the failure and nonfailure groups is summarized in Table 3. For the preoperative parameters, significant differences between the two groups were observed only in SVA (mean ± SD, nonfailure/failure group: − 35.4 ± 26.6/ 34.7 ± 36.4 mm, *p* = 0.002) and PI-LL (− 5.7 ± 14.8/23.0 ± 22.4°, *p* = 0.008), and no significant differences were observed in C-SVA, C2–7A, and T1S. Similarly for the postoperative parameters, significant differences between the two groups were observed in only SVA (4.4 ± 35.2/48.7 ± 29.3 mm, *p* = 0.003) and PI-LL (− 3.3 ± 14.7/21.3 ± 23.4°, *p* = 0.003), and no significant differences were observed in C-SVA, C2–7A, and T1S.

**Table 3:** Comparison of sagittal alignment parameters between the failure and nonfailure groups

	Preoperative			Postoperative		
	Nonfailure (9 cases)	Failure (6 cases)	*P* value	Nonfailure (9 cases)	Failure (6 cases)	*P* value
C-SVA	62.4 ± 15.5	64.3 ± 13.6	0.95	31.1 ± 18.4	39 ± 18.2	0.26
C2–7A	−43.1 ± 27.3	−53.5 ± 16.7	0.78	14.9 ± 10.1	15.8 ± 17.8	0.95
T1slope	33.2 ± 12.8	34.2 ± 19.5	0.95	36.8 ± 10.2	38.5 ± 15.5	1
SVA	**−35.4 ± 26.6**	**34.7 ± 36.4**	0.002*	**4.44 ± 35.2**	**48.7 ± 29.3**	0.0025*
TK	43.2 ± 12.8	35.5 ± 17.3	0.33	41.8 ± 11.0	37.2 ± 16.2	0.33
LL	52.9 ± 11.4	30.3 ± 29.5	0.18	51.7 ± 11.3	30.8 ± 30.1	0.27
PI-LL	**−5.7 ± 14.8**	**23 ± 22.4**	0.008*	**− 3.3 ± 14.7**	**21.3 ± 23.4**	0.003*
PT	20.4 ± 12.7	33.2 ± 11.5	0.09	18.1 ± 11.3	30 ± 13.5	0.11

*Significantly different

**Fig. 2:**
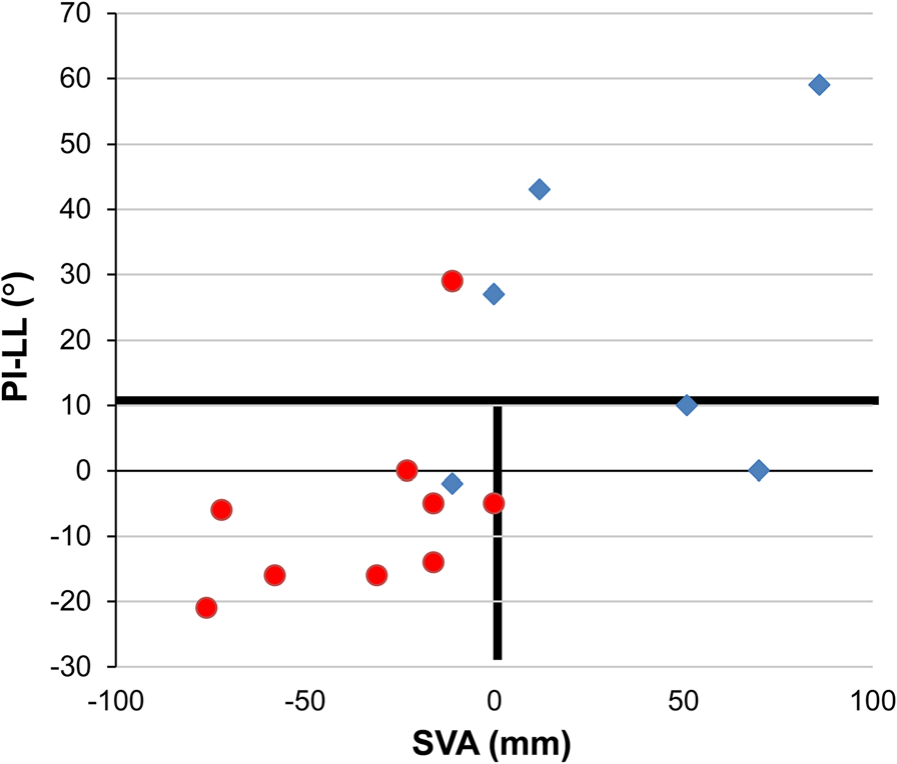
The relationship between SVA (X-axis) and PI-LL (Y-axis). Each plot represents a case (circular plots: nonfailure cases, rhomboid plots: failure cases). SVA: sagittal vertical axis, PI-LL: pelvic incidence minus lumbar lordosis

Regarding the association between the significant sagittal alignment parameters (SVA and PI-LL) and the reasons of additional surgery, high preoperative PI-LL (> 10) patients tended to have lumbar corrective surgery or recommendation after the first operation (3/4, 75%, all additional surgery were lumbar surgery), whereas patients with acceptable PI-LL but positive SVA (> 0 mm) patients (2/2, 100%) had revision surgery for distal junctional failure (Fig. 2).

## Discussion

Our results demonstrated that pre- and postoperative SVA and PI-LL were associated with surgical outcomes of patients with DHS, whereas the preoperative cervical alignment parameters and the corrections of these parameters were not significantly different between the patients with and without treatment failure. Regarding preoperative alignments, negative SVA and normal PI-LL were associated with favorable outcomes. To the best of our knowledge, this is the largest series with clinical outcomes of patients who underwent surgical treatment for DHS to date. In addition, we believe that this is the first study to report the associations between surgical outcomes and global spinal alignments in DHS patients.

Previous studies on surgical treatment for DHS have mostly been single case reports or small case series [11–13, 19]. Even in the recently published systematic review of DHS, only 16 surgically treated cases reported in 15 articles were included [20]. The majority of these reports mainly focused on surgical techniques for the correction and changes in cervical alignment [21, 22], and only a few studies assessed the changes in global spinal alignment in detail [15]. Koda et al. have reported two DHS cases showing an improvement in lower back pain caused by hyperlordosis in the lumbar spine as compensation for head dropping after cervical corrective surgery [23]. Mizutani et al. have described the relationship between cervical spine kyphosis deformities, including DHS, and the sagittal alignment of the global spine and suggested the importance of considering the pathology based on SVA and PI-LL [24], similar to our study. They also reported postoperative cervical and global alignment changes [25]. However, their study did not exclude deformities due to neuromuscular disorders or iatrogenic causes, whereas our study exclusively targeted severe DHS with chin-on-chest deformity and carefully excluded any secondary causes of cervical deformity. Moreover, they did not clearly mention the longitudinal clinical outcomes except alignment changes and seemingly short-term complications/symptoms. Therefore, the impact of these alignment parameters on the hard outcomes of surgical treatment, such as revision surgery, remained unclear.

In our study, patients with horizontal gaze difficulty caused solely by thoracolumbar deformity were carefully excluded. However, the results showed that preoperative PI-LL was still a significant factor for additional surgery. Moreover, postoperative PI-LL was significantly worse in the failure group. Among the patients with abnormal preoperative PI-LL (> 10), the prevalent additional surgeries were thoracolumbar corrective surgeries. To compensate for chin-on-chest deformity in the distal spinal levels, the lumbar spine should be hyperlordotic and PI-LL tends to be smaller or negative. Patients with DHS with high PI-LL are likely to have a dysfunctional compensation in the distal spinal levels. These results suggest that the preoperative PI-LL mismatch is indicative of an independent issue in the lumbar spinal levels and cannot be corrected with cervical corrective surgeries alone. In other words, a significant proportion of patients with DHS, a seemingly cervical issue, have masked clinically significant thoracolumbar deformities as well, which might require corrective surgery. Based on these findings, we believe that the assessment of thoracolumbar alignment parameters should be warranted for all surgical candidates of DHS.

Our results also demonstrated that pre- and postoperative SVA was significantly greater in the failure group. Recently, Hashimoto et al. have reported details on the global sagittal alignment in 20 patients with DHS and discussed changes in alignment for 9 surgical cases. They proposed that DHS could be classified into two groups: SVA+ and SVA− [15]. In the SVA− group, patients maintain compensatory function, including making the lumbar spine hyperlordortic, and can shift the load axis backward. In the SVA+ group, compensatory functions in the thoracolumbar spine and pelvis to drop head deformity are compromised. The results of their study, which highlighted the importance of global sagittal balance and compensatory function in DHS, were compatible to our results. However, the major limitation of their study is that SVA+ patients are heterogeneous and the underlying pathologies, which might affect the surgical outcome, cannot be differentiated only by SVA. We believe that using another sagittal balance parameter (PI-LL) in combination with SVA would be beneficial for patient stratification. Among our patients with normal PI-LL, 100% (2/2) of SVA+ patients in their definitions required multiple revision surgery due to distal junctional failure. Those patients cannot maintain appropriate global spinal alignment and are likely to have postoperative junctional failure due to stress concentration on the implant even after seemingly successful cervical corrective surgeries, which achieved horizontal gaze at least temporarily after surgery.

**Fig. 3:**
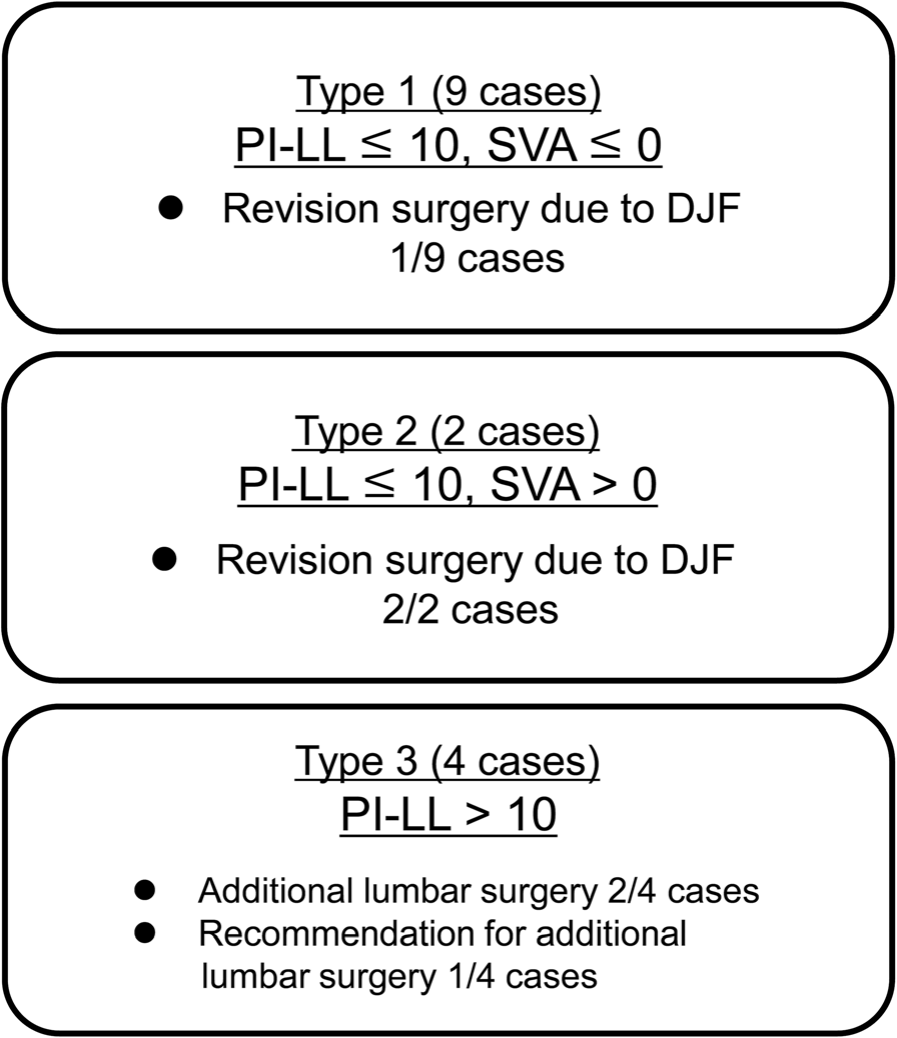
The classification based on two global sagittal alignment parameters and clinical outcomes. SVA: sagittal vertical axis, PI-LL: pelvic incidence minus lumbar lordosis

**Fig. 4:**
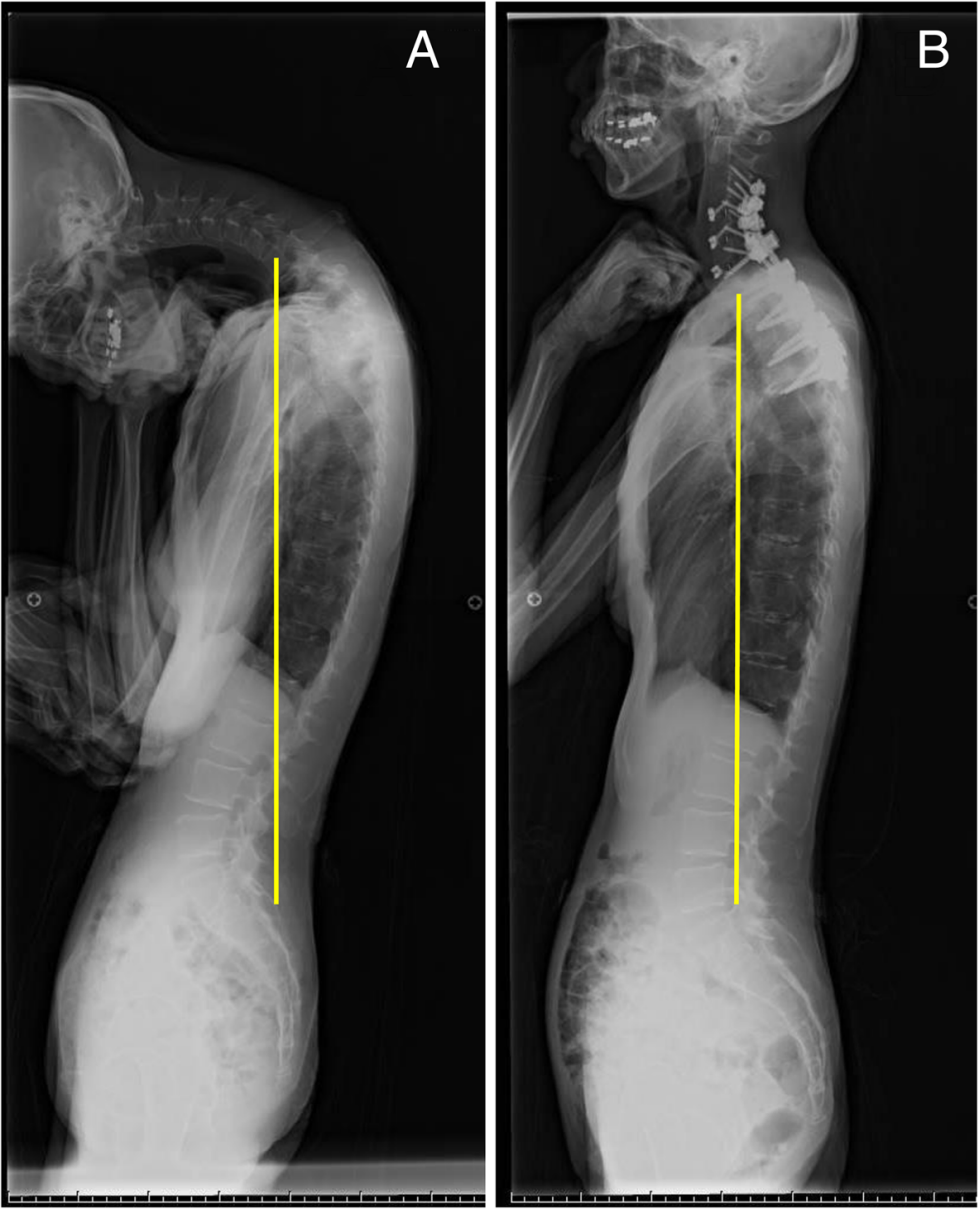
A representative case of type1 . C7 plum line is drowned in each figure. **a** Preoperative lateral whole-spine standing radiograph showing chin-on-chest deformity and SVA of − 23 mm and PI-LL of 0°. **b** Lateral whole-spine standing radiograph at 2 years after the surgery showing well-maintained correction. SVA: sagittal vertical axis, PI-LL: pelvic incidence minus lumbar lordosis

**Fig. 5:**
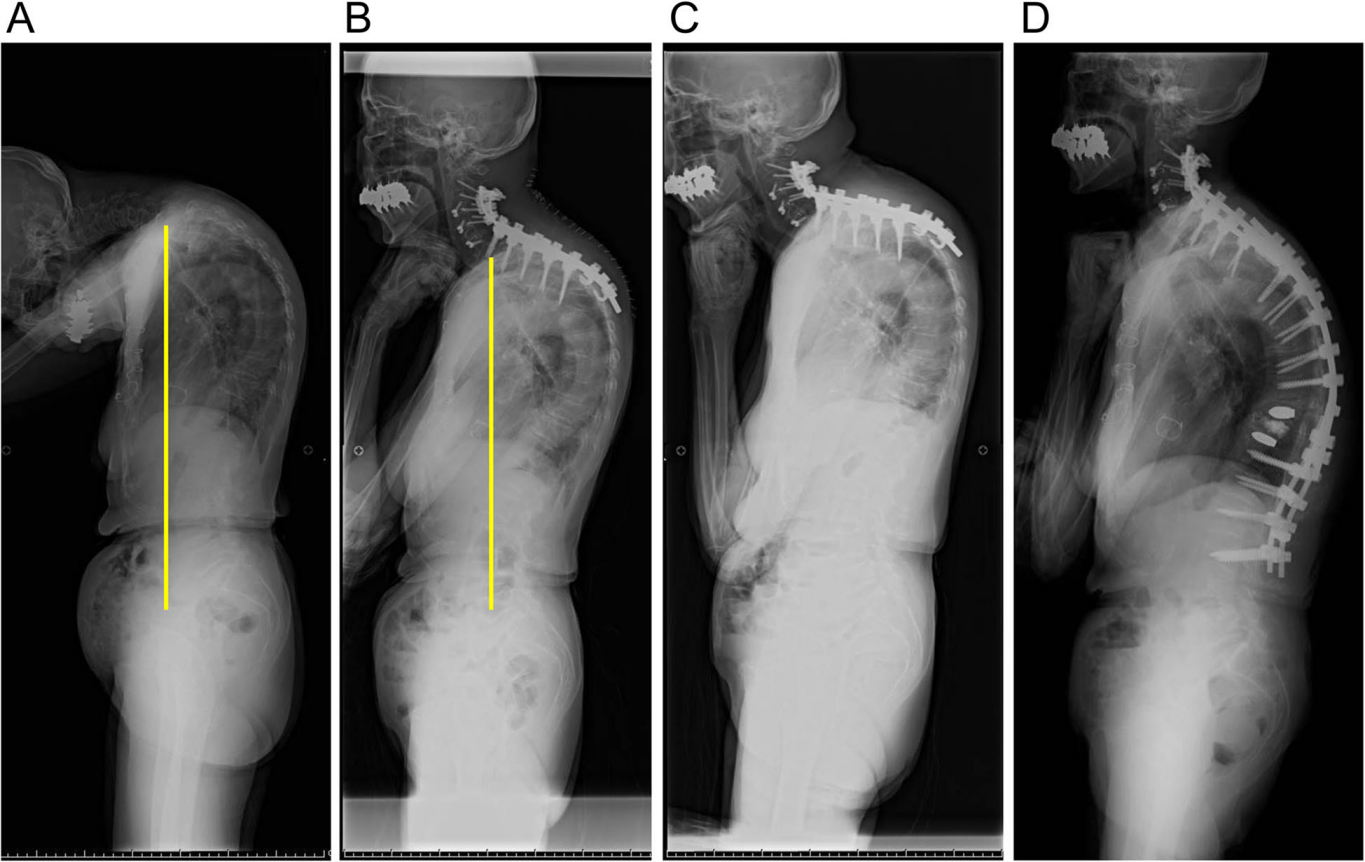
A representative case of type2. C7 plum line is drowned in each figure. **a** Preoperative lateral whole-spine standing radiograph showing chin-on-chest deformity and SVA of 70 mm and PI-LL of 0°. **b** Lateral whole-spine standing radiograph at 2 weeks after the C2-T5 combined anteroposterior corrective fusion showing improvement in chin-on-chest deformity, but the positive SVA (56 mm) remained. **c** Lateral whole-spine standing radiograph at 4 weeks after the initial surgery showing a recurrent deformity due to distal junctional failure. **d** Lateral whole-spine standing radiograph at 12 weeks after the surgery. The patient eventually required a fixation extended down to L2. SVA: sagittal vertical axis, PI-LL: pelvic incidence minus lumbar lordosis

**Fig. 6:**
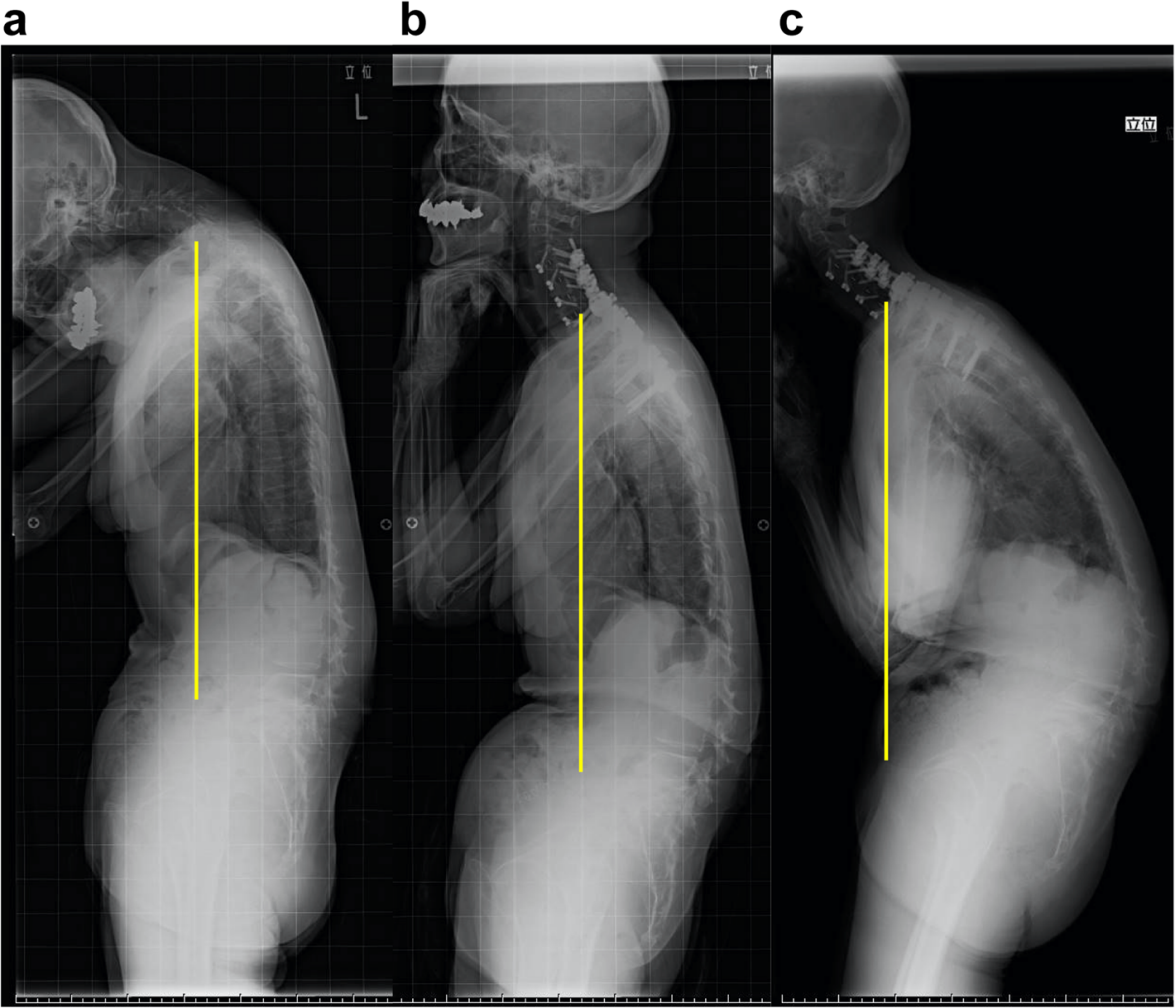
A representative case of type3. C7 plum line was drowned in each Fig. **a** Preoperative lateral whole-spine standing radiograph showing chin-on-chest deformity along with spinopelvic sagittal malalignment. Preoperative parameters were as follows; SVA 86 mm, PI-LL 59°, TK 17°, LL -8°, PT 52°. **b** Lateral whole-spine standing radiograph at 1 month after the C3-T4 combined anteroposterior corrective fusion showing improvement in chin-on-chest deformity, but the thoracolumbar malalignment remained. Postoperative parameters were as follows; SVA 85 mm, PI-LL 60°, TK 23°, LL -9°, PT 53°. **c** Lateral whole-spine standing radiograph at final follow up (36 months after surgery) showing deterioration of a global sagittal balance. We proposed additional thoracolumbar correction surgery for improving low back pain and the recurrence of horizontal gaze disturbance, the medical condition of the patient did not allow additional surgery. Each parameters at final follow up were as follows; SVA 150 mm, PI-LL 75°, TK 25°, LL -24°, PT 60°. SVA: sagittal vertical axis, PI-LL: pelvic incidence minus lumbar lordosis, TK: thoracic kyphosis, LL: lumbar lordosis, PT: pelvic tilt

Based on our results and discussion, patients with DHS could be classified into three categories (Types 1–3, Fig. 3) using two reference points of global spinal sagittal parameters: 1) SVA > 0 mm as proposed by Hashimoto et al. and 2) well-established reference of spinopelvic harmony as PI-LL > 10° [26]. Type 1 is defined as patients with PI-LL of ≤10° and SVA of ≤0 mm (9 patients: Cases 1, 4, 5, 6, 7, 9, 10, 14, and 15, Fig. 4). The compensatory function works well in this type and patients can increase lumbar lordosis enough to shift to SVA−. In our study, the outcomes of this type were favorable except for 1 patient who required an additional surgery, extending the fusion to T4, because of loosening of pedicle screws and delayed-onset minor surgical site infection. All the patients were successfully treated with limited fusions between the cervical and upper thoracic spine. Type 2 is defined as patients with PI-LL of ≤10° and SVA of > 0 mm (2 patients: Cases 2 and 8, Fig. 5). In this group, compensatory function of the spine does not work sufficiently because of concomitant thoracic deformities, including hyperkyphosis, and patients are unable to shift the vertical axis backward. In our study, 2 patients experienced implant failures repeatedly and ultimately required fixation to the lumbar spine. Lastly, Type 3 is defined as patients with PI-LL of > 10° (4 patients: Cases 3, 11, 12, and 13, Fig. 6). These patients have clinically significant thoracolumbar deformity masked by cervical deformity. Even if horizontal gaze is achieved once after cervical surgery, lumbar corrective surgeries are likely to be required for recurrence of horizontal gaze difficulty and/or lumbar spine symptoms.

**Fig. 7:**
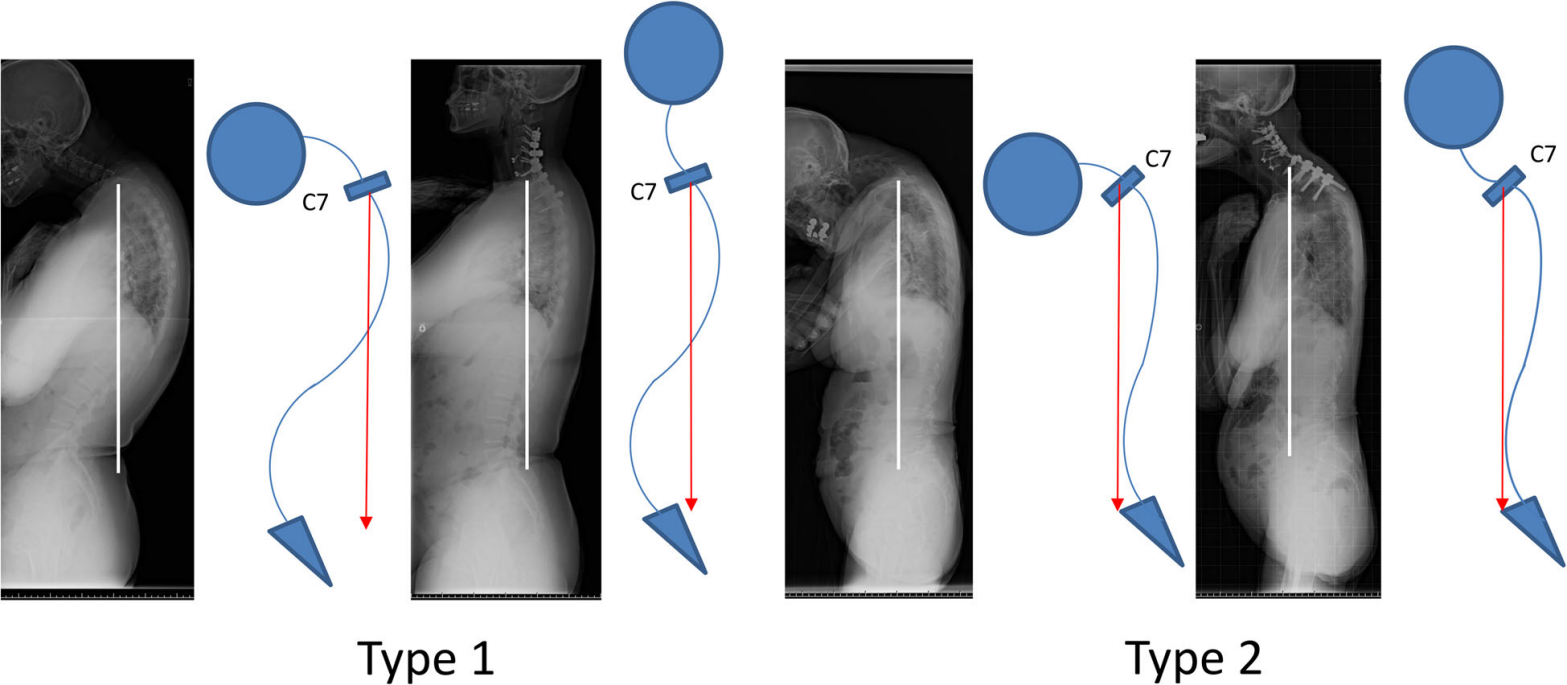
Pre- and post-operative lateral whole-spine standing radiograph and the shame of the patient of Type 1 (Case 10) and Type 2 (Case 2). C7 plum line is drowned in each figure

Regarding the range of fused levels, Sharan et al. have recommended fixation from C2 to the upper thoracic spine T3–5 [1]. However, it is impossible to apply a unified criterion for all patients. In our study, most of the Type 1 patients obtained horizontal gaze and favorable outcomes with fixation extended to the upper thoracic vertebrae, whereas 2 patients in Type 2 eventually required extent of fixation down to the lumbar spine. Among these patients, fixation above the upper thoracic levels appears not enough to shift SVA backward. PI-LL in these patients is normal; therefore, a longer range of fused levels should be considered for correction at thoracic spine level at the first surgery. (Fig. 7) For Type 3 patients, it is difficult to determine the optimal fused range solely by the cervical factors, and the surgical decision-making should be based on both cervical and lumbar pathology assessments.

There are no clear target values of correction of chin-on-chest deformities, similar to PI-LL of < 10° proposed by SRS classification for lumbar kyphosis. In this study, no significant difference was detected in C2–7A, C-SVA, and T1S between the failure and nonfailure groups, suggesting that the cervical spine alignment parameter alone is not enough to predict favorable outcomes. It also depends on the thoracolumbar spinal alignment and compensatory function whether the correction of the cervical spine is sufficient to shift SVA backward. To determine the target values for DHS correction surgery, thoracolumbar spine alignment should be taken into account along with cervical spine alignment to improve outcomes. With our current data, we could not provide definitive values and this point should be addressed in future studies.

Because of the rarity of DHS, our study has several limitations. The first and main limitation is the small sample size. Although our study comprised the largest sample size as an outcome evaluation of surgically treated DHS, the number of patients was not enough to conduct robust statistical analyses. Moreover, the mean follow-up period in this study was approximately 2 years, and the long-term outcomes are yet to be determined. Furthermore, to manage the potentially heterogeneous nature of DHS, surgical techniques were not standardized. Lastly, the main outcome measure of this study was the requirement of revision surgery. Other patient reported outcomes such as pain or ADL scores were not included. We proposed a classification system in this study and we believe that this can be a basis of future study. However, since our study contains several limitations, this classification system should be refined as the evidence grows.

## Conclusion

The comparative analyses between the failure and nonfailure groups showed significant differences in pre- and postoperative PI-LL and SVA. Although chin-on-chest deformity in the cervical spine is a defining feature of DHS, our results suggest that global sagittal alignment parameters, including PI-LL and SVA that likely represent the compensatory function of the thoracic–lumbosacral spine and the displacement in the load axis might have notable impacts on surgical outcomes. Surgeons should pay attention to and consider PI-LL and C7SVA while evaluating each patient’s compensatory function to determine surgical plans for patients with DHS.

## Data Availability

The datasets used and/or analyzed during the current study available from the corresponding author on reasonable request.

## Abbreviations

DHS: Dropped head syndrome
C2–7A: C2–7 angle
C-SVA: C2–7 sagittal vertical axis
T1S: T1 slope
SVA: C7-sacral SVA
TK: Thoracic kyphosis
LL: Lumbar lordosis
PT: Pelvic tilt
PI: Pelvic incidence
HRQOL: Health-related quality of life
